# Generalized external synchronization of networks based on clustered pandemic systems—The approach of Covid-19 towards influenza

**DOI:** 10.1371/journal.pone.0288796

**Published:** 2023-10-12

**Authors:** Muhammad Marwan, Maoan Han, Rizwan Khan

**Affiliations:** 1 School of Mathematical Sciences, Zhejiang Normal University, Jinhua, China; 2 Key Laboratory of Intelligent Education Technology and Application of Zhejiang Province, Zhejiang Normal University, Jinhua, China; Institute of Space Technology, PAKISTAN

## Abstract

Real-world models, like those used in social studies, epidemiology, energy transport, engineering, and finance, are often called “multi-layer networks.” In this work, we have described a controller that connects the paths of synchronized models that are grouped together in clusters. We did this using Lyapunov theory and a variety of coupled matrices to look into the link between the groups of chaotic systems based on influenza and covid-19. Our work also includes the use of external synchrony in biological systems. For example, we have explained in detail how the pandemic disease covid-19 will get weaker over time and become more like influenza. The analytical way to get these answers is to prove a theorem, and the numerical way is to use MATLAB to run numerical simulations.

## 1. Introduction

It was the beginning of 20^*th*^ century, when world encountered with the deadly virus—influenza, that engulfed many precious lives. In literature it is proved that the main cause of its origin was the H1N1 virus with the genes of avian. However, its place of origin is still a paradox but in 1918 it was found in a US military personal. The study of influenza with various perspectives is still of great interest among researchers.

In 2021, Ghanbari and Gómez-Aguilar [1] analysed two influenza dynamical models in the presence of fractional derivative. The study of flu in sea birds was carried out by O’Regan *et al.* [2] in 2013 by considering a perturbed parameter in SIR model. The flu models are not restricted to continuous time systems, Alshaikh [3] worked on the flu model, with respect to constraints of two strains and vaccination, with the aid of forward difference method by discretizing a continuous time model. In 2018, Roberts *et al.* [4] proposed and analysed an influenza-like system for the existence of bifurcation and chaos. A simple SIR model has been converted into SEIR-based model and its dynamics has been discussed by Rezapour *et al.* [5] with the aid of fractional-order derivatives. The influenza model [2] that we have considered in this paper is

$$\begin{array}{cc} {\dot{x}}_{1}= & \omega \left(x_{2}+x_{3}\right)+\omega_{1}x_{2},\\ {\dot{x}}_{2}= & \beta x_{1}x_{2}+\bar{\alpha }x_{2},\\ {\dot{x}}_{3}= & \alpha x_{2}-\omega x_{3}, \end{array}$$ (1)

where $x_{1}$, $x_{2}$, $x_{3}$ are the numbers of susceptible, infected and recovered individuals, respectively.

**Fig 1:**
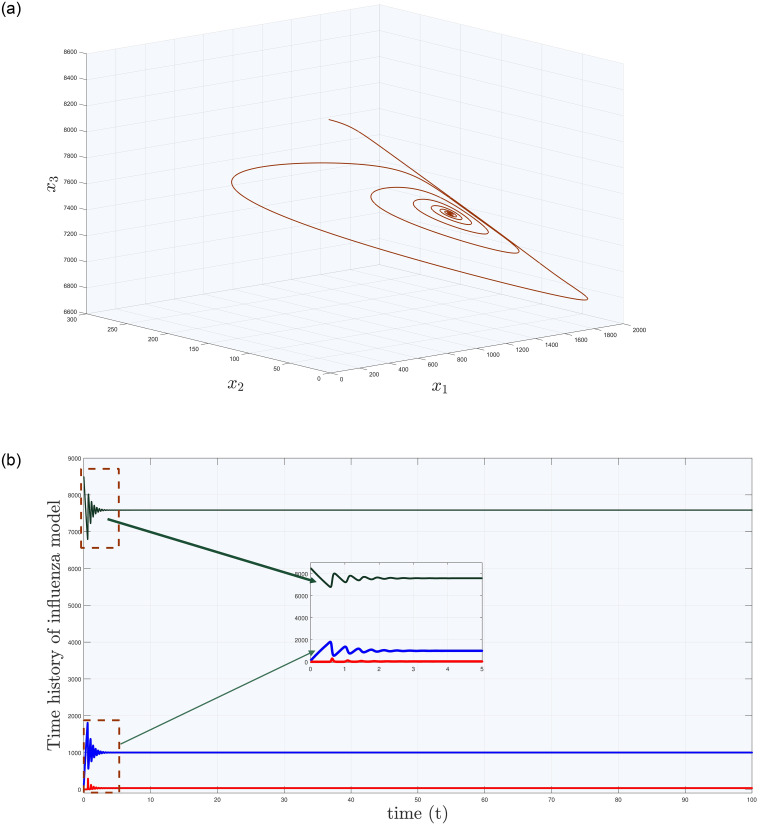
(a) Phase portrait & (b) time history of influenza model (1).

Fig 1 shows the phase portrait and time history of influenza model with $x_{1}=100$, $x_{2}=20$, $x_{3}=8500$ and parameter values given in Table 1. In Fig 1, one can observe that there exist some oscillations, which settles down with the passage of time. From medical point of view, one can consider this situation in a sense that flu is harmful but not that much as was in the past.

**Table 1:** Parameter values of influenza network (5).

parameters	values
*α*	91.25
*β*	0.0924
*ω*	0.4
*ω*_1_	0.75
$\bar{\alpha }$	*α* + *ω* + *ω*_1_

The most recent pandemic that acted similar attitude, begin in the end of 2019 in Wuhan, China. The emergence of this virus has alerted the world and due to globalization its spread was even more faster than the previous diseases. Infinitely, many researchers have worked on covid within their own domain and field. Moreover, the research on covid-19 is not restricted to biology, but a lot of work can be found in other fields as well, such as, Machine learning [6, 7], dynamical systems [8, 9]. The covid-19 model [10, 11] that we have considered is

$$\begin{array}{cc} {\dot{y}}_{1}= & a_{1}y_{3}-a_{2}{\left(y_{3}\right)}^{2}+a_{3}y_{3}y_{2}-a_{4}y_{1}+a_{5}y_{1}y_{3}-a_{6}y_{1}y_{2},\\ {\dot{y}}_{2}= & b_{1}y_{2}y_{3}-b_{2}y_{1}y_{2},\\ {\dot{y}}_{3}= & c_{1}y_{3}-c_{2}y_{1}y_{3}-c_{3}y_{1}y_{2}+c_{4}{\left(y_{1}\right)}^{2}, \end{array}$$ (2)

where $y_{1}$, $y_{2}$ and $y_{3}$ shows the daily numbers of new cases, daily additional severe cases and new death cases reported per day due to covid, respectively. Mathematically, system (2) exhibits chaotic behaviour for initial conditions $\left(y_{1},y_{2},y_{3}\right)$=(184, 30, 8) and parameter values given in Table 2.

**Table 2:** Parameter values of covid-19 network (6).

Parameters	Values	Parameters	Values Values
*a*_1_	66	*b*_1_	0.05507
*a*_2_	1.6966	*b*_2_	0.0008238
*a*_3_	0.148	*c*_1_	0.31303
*a*_4_	0.8763	*c*_2_	0.0001057
*a*_5_	0.022843	*c*_3_	1.008 × 10^−5^
*a*_6_	0.0017342	*c*_4_	1.734 × 10^−6^

**Fig 2:**
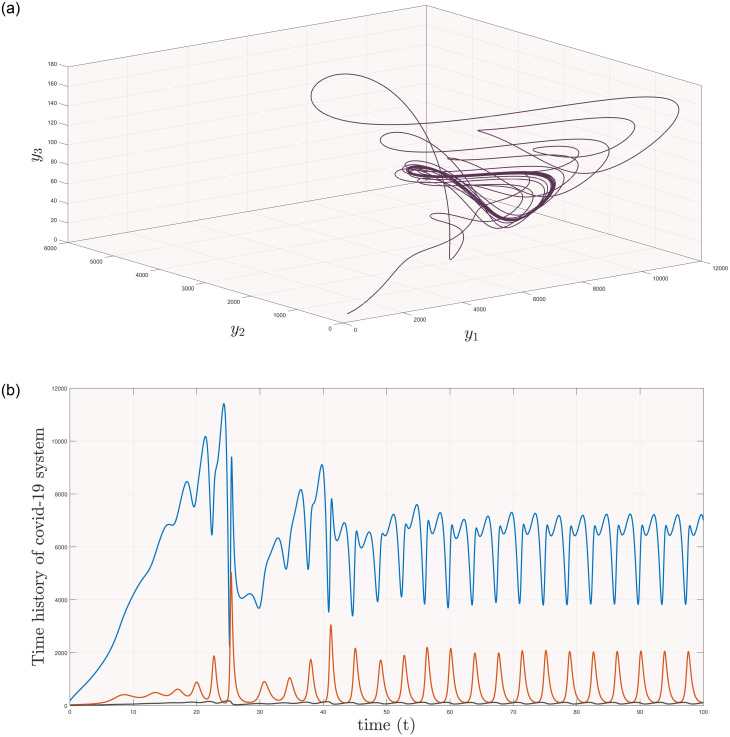
(a) Phase portrait & (b) time history of covid-19 model (2).

Fig 2 shows the phase portrait and time history of covid-19 with $y_{1}=850$, $y_{2}=10$, $y_{3}=8000$ and parameter values given in Table 2. In Fig 2, one can see highly oscillated trajectories leading to unpredictable solution. A set composed of one or more than one ordinary differential equations is known as dynamical system, but the property of sensitivity to its initial conditions and parameter values gave birth to Chaos. Since 1963, uncountable work on chaos can be found such as (Rucklidge [12], Hyperjerk [13], Forced Chen [14], Transportation [15]) systems. In the beginning it was also linked with the butterfly effect. Several researchers used chaos as a positive term in their fields and have showed variety of its applications such as in engineering [16, 17], secure communication [18, 19], biology [20, 21] and reference therein. But, in comparison with the importance of human lives, the term chaos is more dangerous. Keeping in mind its disadvantages, the term controller is designed for such systems, which acted as an external parameter and helps in bringing stability during high oscillations. In the beginning of 1990*s* the term controller is used for the first time in synchronization [22] in which one system termed as ‘slave or derive’ follows the trajectories of other systems called as ‘master’. Many types of synchronization are designed so far, including (complete, generalized, cluster, anti, projective) synchronizations.

In 2000, Josic [23] brought modification into Fenichel for the transverse stability of synchronization manifolds. A simple case study was considered in 2019 for the direct synchronization [24] in two famous Lorenz and Rossler chaotic systems. Lahav *et al.* [25] bring the synchronization between the topology of chaotic systems using various control techniques. In 2021, Majid Hussain considered two integrated time delayed dynamical systems and sorted out the existence of synchronization using the concept of external observer [26], whereas when the trajectories are synchronized with each other in a manner that slave system un-follow the path of master system and shows symmetry in same direction is called anti-synchronization [27, 28].

Network is first studied in the field of mathematical graph theory, which was developed on the base of nodes or vertices that are combined with each other through links or edges. But, now-a-days this topic is of high interest for researchers in the field of dynamical systems, which provides a generic platform for qualitative structures of various systems. However, our interest is the study of such networks in chaos, where finite number of states of chaotic systems can be observed as a network. The state variables $x_{i}$; *i* = 1, 2, ⋯, *n* of each system will be considered as a node and the transitions between these states will be represented as edges. In 2003, Belykh *et al.* [29] derived a technique for the clustering of non-identical systems, whereas Zhongjun [30] has considered connected chaotic networks to for the derivation of clusters among systems. Since the introduction of cluster synchronization and comparison with networks, it has been used in variety of fields, as an application, other than mathematics as well. In 2004, Qin and Chen [31] proposed a technique for the cluster synchronization in the system based on Josephson equations. The investigation of clustered synchronization with non-identical nodes, using pinning control scheme is presented by Lu-Yi-Ning [32]. In 2019 [33], the influence of external network with different densities on a multilayer networks is analysed numerically. Fabio *et al.* worked on the computation of symmetries in multilayer networks [34] which helped in the emergence of cluster synchronization. In time delayed networks under the restriction of leakage, Jayanthi *et al.* [35] designed an algorithm in which the clusters are manipulated to achieve synchronization with respect to isolated node.

Due to the advancement in technology, research publication speed is also increased. Hence, there are a lot of research work that can be found on covid and synchronization but up-to our knowledge from literature survey and above citations, none of the work has been found on the generalized synchronization in which the whole network of chaotic systems is being synchronized to other identical or non-identical network. The current work is being carried out with two main purposes for:

- Engineers: The connection between two clusters under the influence of external controller.
- Biologists: The future prediction about covid-19 and its approach towards influenza.

Hence, our target is to design a suitable controller between two non-identical clusters to achieve synchronization in their trajectories and use it as a tool in biological (covid and influenza) models to show that covid will adopt many variants and will become weaker up to an extent that at the end it will be treated as simple influenza. To achieve this task mathematically, we have adopted the concept of synchronization, because of the nature that, one system can follow path of the other as time advances. In the similar fashion it is proved that covid will follow the path of influenza with the passage of time. Furthermore, we have not restricted our work to single master and slave system, instead we have used the concept of networks in the form of clusters. In other-words, it can be proved that covid-19 is a virus which can never be completely vanished but will adopt some kind of a weaker variant and infected person can be recovered within few days.

This paper is assembled in the following pattern: First, we have given basic definitions in section (2) that will help in understanding rest of the paper. In section (3), we have given a theme of generalized synchronization of two networks. In this section, we have also stated and proved a theorem for achieving the synchronization of networks based on the clusters of (influenza and covid-19) systems in accordance with the steps mentioned in block diagram (4). The validation of our analytical results are given in section (3.2), where the detailed discussion about the approach of covid-19 towards influenza is given in linkage with real life. Finally, we have concluded our paper in section (4).

## 2. Basic definitions

First we give some concepts that are used in understanding rest of the paper.

**Definition 1.1** [36] *Let us consider two dynamical systems of the form*

$$\begin{array}{c} \dot{x}=f\left(x\right)\;and\;\dot{y}=g\left(y\right) \end{array}$$ (3)

*with their corresponding initial conditions, then*

$$\begin{array}{c} \varepsilon =y-\rho x \end{array}$$ (4)

*is the error term between systems in* Eq (3), *where the value of parameter* *ρ* *can be* −1, 0 *or* 1 *depending upon the nature of synchronization* (1.2).

Error term can be used in determining the case and situation that whether at which point function g(y) follows path of another function f(x).

**Definition 1.2** [22] *Let us consider two systems* $\dot{x}$ *and* $\dot{y}$ *given in* Eq (3), *then these systems will be synchronized with each other if the error term* (1.1) *between these systems approaches to zero with the negative definiteness of* $\dot{\nu }$, *where* *ν* *is the energy function* (1.5).

**Definition 1.3** [29] *Suppose there exists a matrix* *A*=[*a*_*lk*_] $\in R^{n\times n}$, *then the matrix A is*:

- *Diffusively Coupled Matrix **(DCM)** if* $\sum_{k=1}^{n}a_{lk}=0$ *for* *l* = 1, 2, ⋯, *n*.
- *Cooperative Coupling if* *a*_*lk*_=*a*_*kl*_ > 0 *for* *l* ≠ *k*.
- *Competitive Coupling if* *a*_*lk*_=*a*_*kl*_ < 0 *for* *l* ≠ *k*.
- *Non Diffusively Coupled Matrix **(NDCM)** if all the off diagonal elements of **DCM** are non-negative*.

The concept of coupled matrices are applicable in the field of control theory, when one can find the existence of clusters and are synchronized within the same system.

**Definition 1.4** [29, 33] *A network consisting of* *n* *nodes exist cluster synchronization, if the total nodes* *n* *divides into some finite clusters, such that the nodes in the same cluster synchronize with each other*.

**Fig 3:**
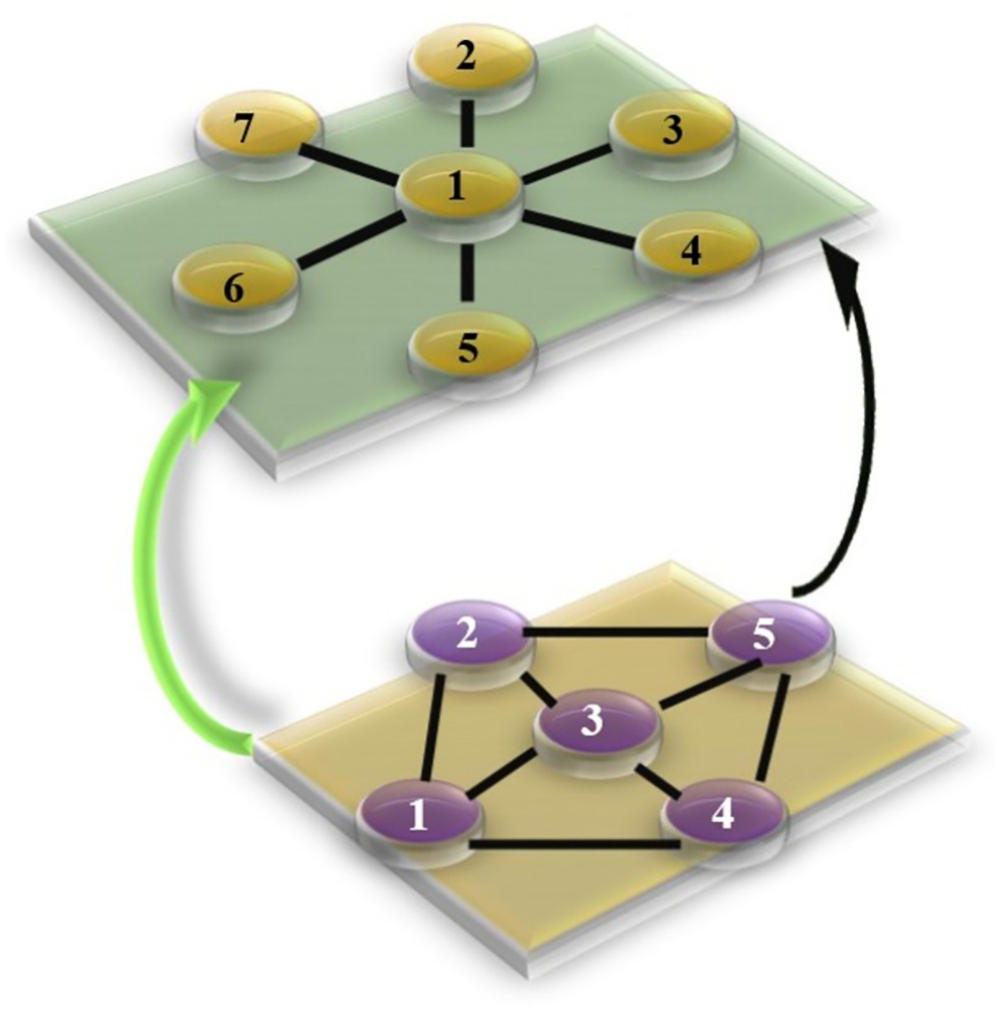
Various types of networks coupled and synchronized with each other.

In Fig 3 we can see the nodes of a network are linked with each other in the various structures.

**Definition 1.5** [36] *A function* ν(x) *is said to be Lyapunov function if it satisfies the following properties*:

-
ν(x)=0 , *for* x=0.
-
ν(x)>0 , *for all* x≠0.

The positive and negative definiteness of a function depends upon the differentiation of a Lyapunov function *ν*.

**Remark 1.6** *If definition* (1.5) *is encountered with the additional property that, If* $\dot{\nu }\left(x\right)<0$ *for all* x≠0 *then, such Lyapunov functions are considered to be negative definite, otherwise are positive definite*.

## 3. Generalized synchronization

**Fig 4:**
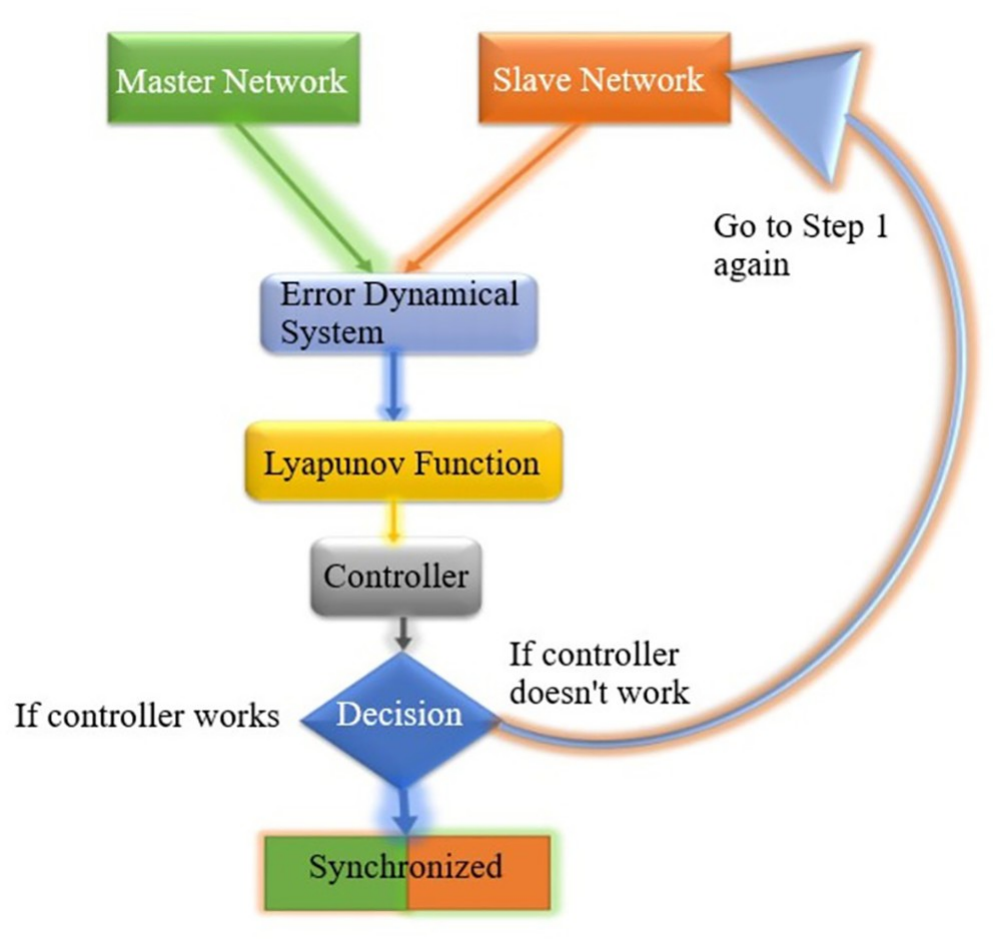
Block diagram for the synchronization of clustered networks.

In this section, we will follow the block diagram given in Fig 4 for the synchronization of two networks based on chaotic systems. Therefore, we consider system of the form;

$$\begin{array}{c} {\dot{x}}_{i}^{m}=f\left(x_{i}^{m}\right)+\epsilon \sum_{j=1}^{n}c_{ij}x_{j}^{m} \end{array}$$ (5)

for *i* = 1, 2, ⋯, *n* as a master network in which *c*_*ij*_ are entry of a coupled matrix (1.3) used for clustered networking and is helpful in synchronizing *n*-identical systems as a network. We consider another network, which can work as a slave network

$$\begin{array}{c} {\dot{y}}_{i}^{s}=g\left(y_{i}^{s}\right)+\epsilon \sum_{j=1}^{n}d_{ij}y_{j}^{s}+U_{ms} \end{array}$$ (6)

for *i* = 1, 2, ⋯, *n* with the clustered matrix *d*_*ij*_. *U*_*ms*_ in Eq (6) is the external controller, which can be designed latter, used for the synchronization of master and slave networks. In both cases the *ϵ* is perturbed parameter and can be selected within the range of (0, 1). Generally, in synchronization and its all types are dependant on the concept of error term (1.1):

$$\begin{array}{c} \varepsilon_{i}=y_{i}^{s}-\rho x_{i}^{m} \end{array}$$ (7)

where *ρ* is generalized parameter and play a role of bridge between synchronization and anti-synchronization. Differentiating Eq (7) to get error dynamical system:

$$\begin{array}{c} {\dot{\varepsilon }}_{i}=h\left(\varepsilon \right)+\epsilon \sum_{j=1}^{n}E_{ij}e_{j}+\text{Extra}\;\text{Terms} \end{array}$$ (8)

for *i* = 1, 2, ⋯, *n*. Our target is to achieve negative definiteness of differentiation of energy function (1.5). We will achieve our target of synchronization with the differentiation of $\dot{\nu }<0$, otherwise we need to go back to first step and repeat.

### 3.1 Generalized synchronization for Influenza and Covid-19 networks

A system of influenza can be re-written in the following form

$$\begin{array}{cc} {\dot{x}}_{i}= & \omega \left(y_{i}+z_{i}\right)+\omega_{1}y_{i}+u_{c},\\ {\dot{y}}_{i}= & \beta x_{i}y+\bar{\alpha }y_{i},\\ {\dot{z}}_{i}= & \alpha y_{i}-\omega z_{i}, \end{array}$$ (9)

for *i* = 1, 2, ⋯, *n* and *u*_*c*_=$\sum_{j=1}^{n}c_{ij}x_{j}$ is the control input used for the creation of clusters in system (9), and *c*_*ij*_ represents the coupled matrix which fulfils the criteria of definition (1.3).

**Remark 2.1** *Let us choose* *u*_*c*_=0 *and* *i* = 1, *then the network* (9) *is reduced to influenza system* (1) *with the phase portraits given in* Fig 1.

**Fig 5:**
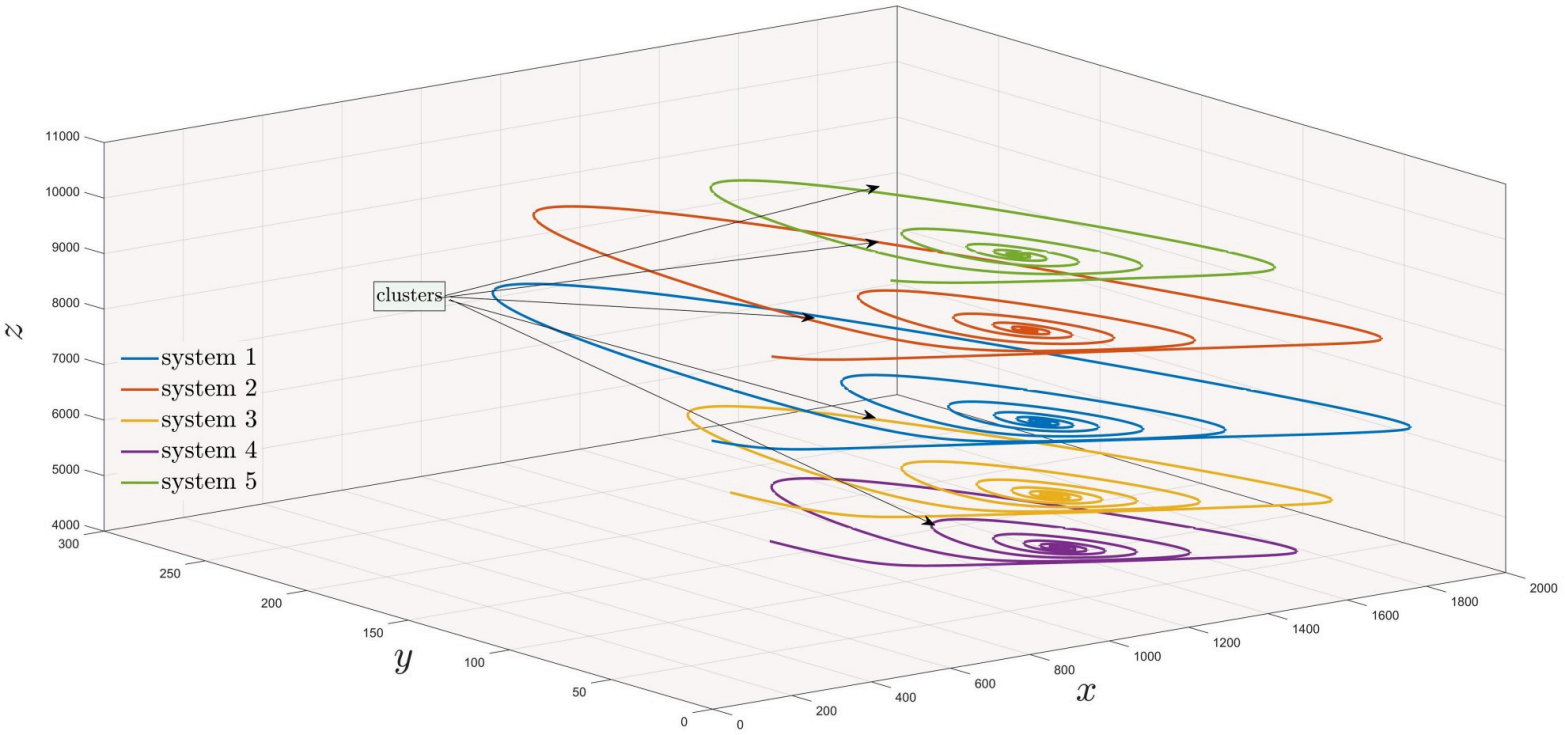
Existence of five influenza models in a cluster.

**Fig 6:**
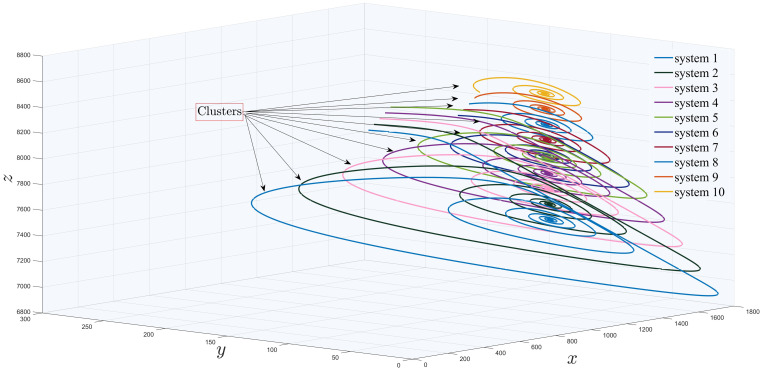
Existence of ten influenza models in a cluster.

In Fig 5, there are five clusters which are synchronized with each other and are plotted against the control input *u*_*c*_=$\sum_{j=1}^{5}c_{ij}^{a}x_{j}$ with

$$\begin{array}{c} C^{a}=(\begin{array}{ccccc} 4 & -4 & 1 & -2 & 1\\ -4 & 4 & -2 & 1 & 1\\ 1 & -1 & 6 & -3 & -3\\ -1 & 1 & -3 & 6 & -3\\ 1 & 1 & -4 & -4 & 6 \end{array}), \end{array}$$ (10)

while Fig 6 is plotted for ten clusters with the control input *u*_*c*_=$\sum_{j=1}^{10}c_{ij}^{b}x_{j}$ with

$$\begin{array}{c} C^{b}=(\begin{array}{cccccccccc} 7 & -7 & 2 & -2 & 0 & 5 & -1 & -1 & -2 & -1\\ -7 & 7 & -2 & 2 & 0 & 2 & -2 & 3 & 1 & -4\\ 2 & -2 & 5 & -2 & -3 & -6 & 2 & 3 & 1 & 0\\ -2 & 2 & -2 & 5 & -3 & 2 & 3 & 1 & 0 & -6\\ 0 & 0 & -3 & -3 & 6 & 8 & -5 & -3 & 0 & 0\\ 5 & 2 & -6 & 2 & 8 & -1 & -3 & -2 & -5 & 0\\ -1 & -2 & 2 & 3 & -5 & 3 & -4 & 2 & 0 & 2\\ -1 & 3 & 3 & 1 & -3 & -2 & -1 & 0 & 1 & -1\\ -2 & 1 & 1 & 0 & 0 & 0 & -3 & -2 & -5 & 0\\ -1 & -4 & 0 & -6 & 0 & 1 & 3 & 2 & 5 & 0 \end{array}). \end{array}$$ (11)

In similar fashion a covid-19 model can be transformed in the following network:

$$\begin{array}{cc} {\dot{p}}_{i}= & a_{1}r_{i}-a_{2}{\left(r_{i}\right)}^{2}+a_{3}r_{i}q_{i}-a_{4}p_{i}+a_{5}p_{i}r_{i}-a_{6}p_{i}q_{i}+u_{c}^{cov},\\ {\dot{q}}_{i}= & b_{1}q_{i}r_{i}-b_{2}p_{i}q_{i},\\ {\dot{r}}_{i}= & c_{1}r_{i}-c_{2}p_{i}r_{i}-c_{3}p_{i}q_{i}+c_{4}{\left(p_{i}\right)}^{2}, \end{array}$$ (12)

for *i* = 1, 2, ⋯, *n* where $u_{c}^{cov}=\sum_{j=1}^{n}d_{ij}x_{j}$ is the clustered control input used with a real valued matrix *D* = [*d*_*ij*_].

**Fig 7:**
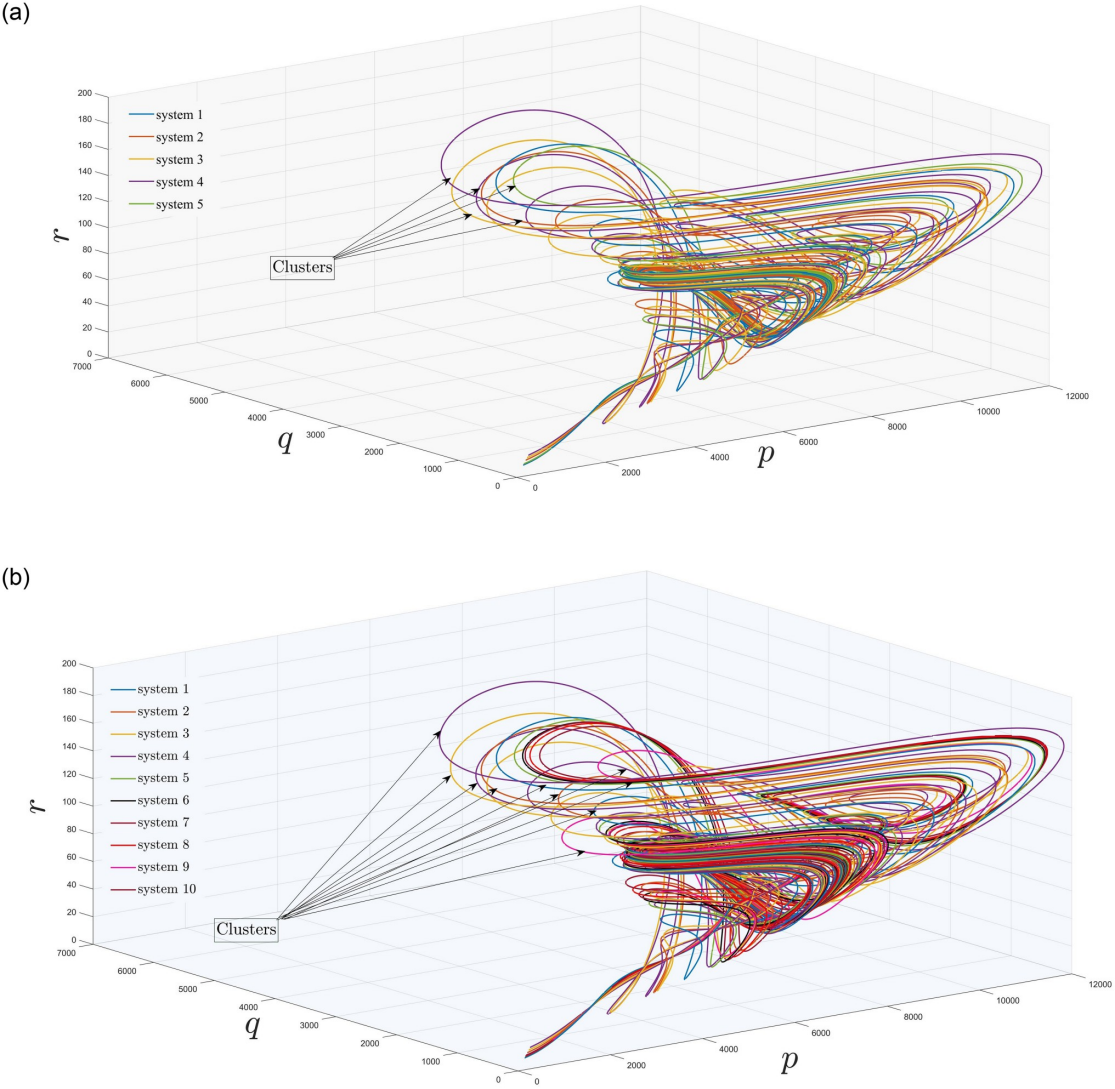
Existence of (a) five and (b) ten, covid-19 models in a cluster.

Fig 7(a) and 7(b) show the cluster of covid chaotic model with *D*^*a*^=*C*^*a*^ and

$$\begin{array}{c} D^{b}=(\begin{array}{cccccccccc} 4 & -4 & 1 & -2 & 1 & 9 & -4 & -2 & -2 & -1\\ -4 & 4 & -2 & 1 & 1 & 2 & 4 & -1 & -1 & -4\\ 1 & -1 & 6 & -3 & -3 & -6 & 3 & 1 & 2 & 0\\ -1 & 1 & -3 & 6 & -3 & 0 & 5 & 1 & 0 & -6\\ 1 & 1 & -4 & -4 & 6 & -3 & -4 & 7 & 0 & 0\\ 9 & 2 & -6 & 0 & -3 & 0 & -1 & -1 & 0 & 0\\ -4 & 4 & 3 & 5 & -4 & 1 & 0 & -2 & -1 & -2\\ -2 & -1 & 1 & 1 & 7 & -1 & -1 & -3 & 0 & -1\\ -2 & -1 & 2 & 0 & 0 & 0 & 0 & 1 & -3 & 3\\ -1 & -4 & 0 & -6 & 0 & 4 & 2 & 4 & 1 & 0 \end{array}) \end{array}$$ (13)

in five and ten subsystems respectively. Now, we are able to use results given in Sect. 2 to synchronize the trajectories of networks based on covid and influenza models.

**Theorem 2.2** *A slave network* (15) *follows the path of master network* (14) *if the error terms between them approaches to zero with the control input* (19).

***Proof:*** We begin our proof with the master influenza network:

$$\begin{array}{cc} {\dot{p}}_{i}^{m}= & \omega \left(q_{i}^{m}+r_{i}^{m}\right)+\omega_{1}q_{i}^{m}+u_{inf},\\ {\dot{q}}_{i}^{m}= & \beta p_{i}^{m}q^{m}+\bar{\alpha }q_{i}^{m},\\ {\dot{r}}_{i}^{m}= & \alpha q_{i}^{m}-\omega r_{i}^{m}, \end{array}$$ (14)

for *i* = 1, 2, ⋯, *n*, where $u_{inf}=\sum_{j=1}^{n}c_{ij}x_{j}^{m}$ and selecting covid-19 as slave network

$$\begin{array}{cc} {\dot{p}}_{i}^{s}= & a_{1}r_{i}^{s}-a_{2}{\left(r_{i}^{s}\right)}^{2}+a_{3}r_{i}^{s}q_{i}^{s}-a_{4}p_{i}^{s}+a_{5}p_{i}^{s}r_{i}^{s}-a_{6}p_{i}^{s}q_{i}^{s}+u_{cov}+\Theta_{1i},\\ {\dot{q}}_{i}^{s}= & b_{1}q_{i}^{s}r_{i}^{s}-b_{2}p_{i}^{s}q_{i}^{s}+\Theta_{2i},\\ {\dot{r}}_{i}^{s}= & c_{1}r_{i}^{s}-c_{2}p_{i}^{s}r_{i}^{s}-c_{3}p_{i}^{s}q_{i}^{s}+c_{4}{\left(p_{i}^{s}\right)}^{2}+\Theta_{3i}, \end{array}$$ (15)

for *i* = 1, 2, ⋯, *n*, where $u_{cov}=\sum_{j=1}^{n}d_{ij}x_{j}^{s}$. Further, Θ_*i*_; *i* = 1, 2, 3 are the control inputs, used for external synchronization, that can be designed latter on the basis of energy type function (1.5). The error dynamical network of master (14) and slave (15) networks, using Eq (7) is:

$$\begin{array}{ccc} {\dot{\varepsilon }}_{1i} & = & a_{1}\varepsilon_{3i}-a_{4}\varepsilon_{1i}-a_{4}\rho p_{i}^{m}+\left(a_{1}-\omega \right)\rho r_{i}^{m}-a_{2}{\left(\varepsilon_{3i}+\rho r_{i}^{m}\right)}^{2}\\ & + & \left(a_{3}\varepsilon_{2i}+a_{5}\varepsilon_{1i}\right)\varepsilon_{3i}+\left(a_{3}\varepsilon_{2i}+a_{5}\varepsilon_{1i}\right)\rho \varepsilon_{3i}\\ & + & \left(a_{3}q_{i}^{m}+a_{5}p_{i}^{m}\right)\rho \varepsilon_{3i}+\left(a_{3}q_{i}^{m}+a_{5}p_{i}^{m}\right)\rho^{2}r_{i}^{m}\\ & - & \rho \left(\omega +\omega_{1}+a_{6}\varepsilon_{1i}\right)q_{i}^{m}-a_{6}\left(\left(\varepsilon_{1i}+\rho p_{i}^{m}\right)\varepsilon_{2i}+\rho^{2}p_{i}^{m}q_{i}^{m}\right)\\ & + & \epsilon \sum_{j=1}^{n}\left(d_{ij}\varepsilon_{1j}+\left(d_{ij}-c_{ij}\right)\rho p_{j}^{m}\right)+\Theta_{1i},\\ {\dot{\varepsilon }}_{2i} & = & \varepsilon_{2i}(b_{1}\varepsilon_{3i}-b_{2}\varepsilon_{1i}+b_{1}\rho r_{i}^{m}-b_{2}\rho p_{i}^{m})-\rho^{2}q_{i}^{m}r_{i}^{m}\\ & + & \rho q_{i}^{m}(b_{1}\varepsilon_{3i}-\bar{\alpha }-b_{2}\varepsilon_{1i})-(\rho^{2}-\rho \beta )p_{i}^{m}q_{i}^{m}+\Theta_{2i},\\ {\dot{\varepsilon }}_{3i} & = & c_{1}\varepsilon_{3i}-(c_{2}\varepsilon_{3i}+c_{3}\varepsilon_{2i})(\rho p_{i}^{m}+\varepsilon_{1i})-(c_{2}r_{i}^{m}+c_{3}q_{i}^{m})\rho^{2}p_{i}^{m}\\ & + & c_{4}{\left(\varepsilon_{1i}+\rho p_{i}^{m}\right)}^{2}+\rho (c_{1}+\omega -c_{2}\varepsilon_{1i})r_{i}^{m}-\rho (c_{3}\varepsilon_{1i}+\alpha )q_{i}^{m}+\Theta_{3i}, \end{array}$$ (16)

for *i* = 1, 2, ⋯, *n*. Now, in order to achieve the control inputs, we have selected

$$\begin{array}{c} \nu \left(\varepsilon \right)=\sum_{k=1}^{3}{\left(\varepsilon_{ki}\right)}^{2};\;i=1,2,\cdots ,n. \end{array}$$ (17)

Lyapunov function, which satisfies the criteria of positive definiteness given in definition (1.5) with *ν*(*ε*) ≥ 0 for all *ε*. Furthermore, to achieve synchronization, we need to show that all the error terms are equal to zero. For this purpose, we have only to prove that the derivative of Lyapunov function; *ν* is negative definite. Hence,

$$\begin{array}{ccc} \dot{\nu } & = & a_{1}\varepsilon_{1i}\varepsilon_{3i}-a_{4}\varepsilon_{1i}^{2}-a_{4}\varepsilon_{1i}\rho p_{i}^{m}+\left(a_{1}-\omega \right)\rho \varepsilon_{1i}r_{i}^{m}-a_{2}\varepsilon_{1i}{\left(\varepsilon_{3i}+\rho r_{i}^{m}\right)}^{2}\\ & + & \left(a_{3}\varepsilon_{2i}+a_{5}\varepsilon_{1i}\right)\varepsilon_{1i}\left(\varepsilon_{3i}+\rho \varepsilon_{3i}\right)+\left(a_{3}q_{i}^{m}+a_{5}p_{i}^{m}\right)\varepsilon_{1i}\left(\rho \varepsilon_{3i}+\rho^{2}r_{i}^{m}\right)\\ & - & \rho \left(\omega +\omega_{1}+a_{6}\varepsilon_{1i}\right)\varepsilon_{1i}q_{i}^{m}-a_{6}\left(\left(\varepsilon_{1i}+\rho p_{i}^{m}\right)\varepsilon_{2i}+\rho^{2}p_{i}^{m}q_{i}^{m}\right)\varepsilon_{1i}\\ & + & \varepsilon_{2i}^{2}(b_{1}\varepsilon_{3i}-b_{2}\varepsilon_{1i}+b_{1}\rho r_{i}^{m}-b_{2}\rho p_{i}^{m})-\rho^{2}\varepsilon_{2i}q_{i}^{m}r_{i}^{m}\\ & + & \rho q_{i}^{m}(b_{1}\varepsilon_{3i}-\bar{\alpha }-b_{2}\varepsilon_{1i})\varepsilon_{2i}-(\rho^{2}-\rho \beta )\varepsilon_{2i}p_{i}^{m}q_{i}^{m}\\ & + & c_{1}\varepsilon_{3i}^{2}-(c_{2}\varepsilon_{3i}+c_{3}\varepsilon_{2i})\varepsilon_{3i}(\rho p_{i}^{m}+\varepsilon_{1i})-(c_{2}r_{i}^{m}+c_{3}q_{i}^{m})\rho^{2}\varepsilon_{3i}p_{i}^{m}\\ & + & c_{4}{\left(\varepsilon_{1i}+\rho p_{i}^{m}\right)}^{2}\varepsilon_{3i}+\rho (c_{1}+\omega -c_{2}\varepsilon_{1i})\varepsilon_{3i}r_{i}^{m}-\rho (c_{3}\varepsilon_{1i}+\alpha )\varepsilon_{3i}q_{i}^{m}\\ & + & \epsilon \sum_{j=1}^{n}\left(d_{ij}\varepsilon_{1j}+\left(d_{ij}-c_{ij}\right)\rho p_{j}^{m}\right)\varepsilon_{1i}+\Theta_{1i}\varepsilon_{1i}+\Theta_{2i}\varepsilon_{2i}+\Theta_{3i}\varepsilon_{3i}. \end{array}$$ (18)

Now, we need to design appropriate control inputs; Θ_1*i*_, Θ_2*i*_, Θ_3*i*_ such that Eq (18) becomes negative definite. Therefore, in view of Eqs (16) and (18) the control inputs are:

$$\begin{array}{ccc} \Theta_{1i} & = & -{ϒ}_{1}\varepsilon_{1i}+a_{4}\rho p_{i}^{m}-\left(a_{1}-\omega \right)\rho r_{i}^{m}+a_{2}{\left(\varepsilon_{3i}+\rho r_{i}^{m}\right)}^{2}\\ & - & \left(a_{3}\varepsilon_{2i}+a_{5}\varepsilon_{1i}\right)\left(\varepsilon_{3i}+\rho \varepsilon_{3i}\right)-\left(a_{3}q_{i}^{m}+a_{5}p_{i}^{m}\right)\left(\rho \varepsilon_{3i}+\rho^{2}r_{i}^{m}\right)\\ & + & \rho \left(\omega +\omega_{1}+a_{6}\varepsilon_{1i}\right)q_{i}^{m}+a_{6}\left(\left(\varepsilon_{1i}+\rho p_{i}^{m}\right)\varepsilon_{2i}+\rho^{2}p_{i}^{m}q_{i}^{m}\right)\\ & - & \epsilon \sum_{j=1}^{n}\left(\left(d_{ij}-c_{ij}\right)\rho p_{j}^{m}\right),\\ \Theta_{2i} & = & -{ϒ}_{2}\varepsilon_{2i}-b_{1}\varepsilon_{2i}\varepsilon_{3i}+b_{2}\varepsilon_{2i}\varepsilon_{1i}-b_{1}\rho \varepsilon_{2i}r_{i}^{m}+b_{2}\rho \varepsilon_{2i}p_{i}^{m}+\rho^{2}q_{i}^{m}r_{i}^{m}\\ & - & \rho q_{i}^{m}(b_{1}\varepsilon_{3i}-\bar{\alpha }-b_{2}\varepsilon_{1i})-(\rho^{2}-\rho \beta )p_{i}^{m}q_{i}^{m},\\ \Theta_{3i} & = & -{ϒ}_{3}\varepsilon_{3i}+(c_{2}\varepsilon_{3i}+c_{3}\varepsilon_{2i})(\rho p_{i}^{m}+\varepsilon_{1i})+(c_{2}r_{i}^{m}+c_{3}q_{i}^{m})\rho^{2}p_{i}^{m}\\ & - & c_{4}{\left(\varepsilon_{1i}+\rho p_{i}^{m}\right)}^{2}-\rho (c_{1}+\omega -c_{2}\varepsilon_{1i})r_{i}^{m}+\rho (c_{3}\varepsilon_{1i}+\alpha )q_{i}^{m}. \end{array}$$ (19)

Putting back Eq (19) into Eqs (16) and (18), we get

$$\begin{array}{c} \left\{ \begin{array}{c} {\dot{\varepsilon }}_{1i}=-\left({ϒ}_{1}+a_{4}\right)\varepsilon_{1i}+a_{1}\varepsilon_{3i}+\epsilon \sum_{j=1}^{n}\left(d_{ij}\varepsilon_{1j}\right)\\ {\dot{\varepsilon }}_{2i}=-{ϒ}_{2}\varepsilon_{2i}\\ {\dot{\varepsilon }}_{3i}=\left(-{ϒ}_{3}+c_{1}\right)\varepsilon_{3i} \end{array}\right. \end{array}$$ (20)

and

$$\begin{array}{c} \dot{\nu }=-\left({ϒ}_{1}+a_{4}\right)\varepsilon_{1i}^{2}+a_{1}\varepsilon_{1i}\varepsilon_{3i}+\epsilon \varepsilon_{1i}\sum_{j=1}^{n}\left(d_{ij}\varepsilon_{1j}\right)-{ϒ}_{2}\varepsilon_{2i}^{2}-\left({ϒ}_{3}-c_{1}\right)\varepsilon_{3i}^{2}. \end{array}$$ (21)

Further, we get:

$$\begin{array}{ccc} \dot{\nu } & \leq & -(\left({ϒ}_{1}+a_{4}\right)\varepsilon_{1i}^{2}+{ϒ}_{2}\varepsilon_{2i}^{2}+\left({ϒ}_{3}-c_{1}\right)\varepsilon_{3i}^{2}) \end{array}$$ (22)

for −(*ϒ*_1_ + *a*_4_) ≥ *a*_1_ and (*ϒ*_3_ > *c*_1_). Eq (22) shows that, the derivative of our considered Lyapunov function is negative definite. Hence, we get our desired result.

The parameter; *ρ* used in our main result (2.2) is the generalized parameter and is playing important role as a bridge between direct and anti synchronizations.

**Remark 2.3** *If we consider* *ρ* = 1 *in Theorem* (2.2), *then direct synchronization between influenza and covid networks will occur. Considering* *ρ* = −1 *in Theorem* (2.2) *will lead to anti-synchronization, whereas there will exist no synchronization for* *ρ* = 0.

This theorem is the analytical result for the synchronization of influenza and covid networks. In next section, we have provided its numerical simulation.

#### 3.2 Discussion on the basis of numerical simulations & real life comparison

In section (3), we have discussed influenza and covid-19 models in sense of networks. In Figs 5–7, we have observed the existence of influenza and covid systems in their corresponding clusters. Moreover, in their own they are synchronized with each other as well with the aid of some suitable controller. After that, we have stated and proved Theorem (2.2) for generalized synchronization of influenza (master) and covid-19 (slave) networks. Now, in this section, we will perform numerical simulation for the validation of our analytical results and will compare with the real life scenario as well. For numerical simulations, we have used MATLAB 2019*b*, using ODE45 for solving our considered models. Moreover, throughout this paper, ([$p_{1}^{m}$, $q_{1}^{m}$, $r_{1}^{m}$], [$p_{2}^{m}$, $q_{2}^{m}$, $r_{2}^{m}$], [$p_{3}^{m}$, $q_{3}^{m}$, $r_{3}^{m}$], [$p_{4}^{m}$, $q_{4}^{m}$, $r_{4}^{m}$], [$p_{5}^{m}$, $q_{5}^{m}$, $r_{5}^{m}$], [$p_{6}^{m}$, $q_{6}^{m}$, $r_{6}^{m}$], [$p_{7}^{m}$, $q_{7}^{m}$, $r_{7}^{m}$], [$p_{8}^{m}$, $q_{8}^{m}$, $r_{8}^{m}$], [$p_{9}^{m}$, $q_{9}^{m}$, $r_{9}^{m}$], [$p_{10}^{m}$, $q_{10}^{m}$, $r_{10}^{m}$]) = ([100, 50, 8500], [200, 60, 8510], [300, 70, 8520], [400, 80, 8530], [500, 90, 8540], [600, 50, 8500], [700, 60, 8510], [800, 70, 8520], [900, 80, 8530], [1000, 90, 8540]) are the initial conditions for network (5) with the parameter values given in Table 1.

whereas ([$p_{1}^{s}$, $q_{1}^{s}$, $r_{1}^{s}$], [$p_{2}^{s}$, $q_{2}^{s}$, $r_{2}^{s}$], [$p_{3}^{s}$, $q_{3}^{s}$, $r_{3}^{s}$], [$p_{4}^{s}$, $q_{4}^{s}$, $r_{4}^{s}$], [$p_{5}^{s}$, $q_{5}^{s}$, $r_{5}^{s}$], [$p_{6}^{s}$, $q_{6}^{s}$, $r_{6}^{s}$], [$p_{7}^{s}$, $q_{7}^{s}$, $r_{7}^{s}$], [$p_{8}^{s}$, $q_{8}^{s}$, $r_{8}^{s}$], [$p_{9}^{s}$, $q_{9}^{s}$, $r_{9}^{s}$], [$p_{10}^{s}$, $q_{10}^{s}$, $r_{10}^{s}$]) = [184308], [257.6, 42, 11.2], [294.4, 48, 12.8], [331.2, 54, 14.4], [202.4, 33, 8.8], [206.08, 33.6, 8.96], [209.76, 34.2, 9.12], [213.44, 34.8, 9.28], [217.12, 35.4, 9.44], [220.8, 36, 9.6] are the initial conditions for network (6) with the parameter values given in Table 2.

**Fig 8:**
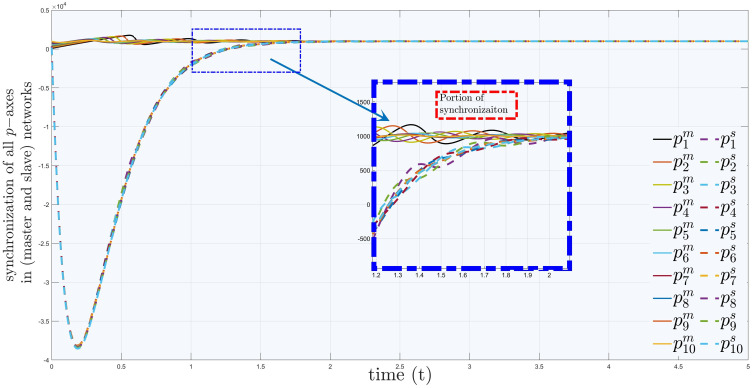
Synchronization of *p*−*axis* in master and slave networks.

Fig 8 is the time history of synchronization in *p*−axis directions of influenza and covid-19 networks. In this figure, we have highlighted a region by bounding in a blue coloured rectangle, where synchronization takes place and all *p*−axis trajectories of covid-19 network started following the same axes of influenza network at *t* = 1.5.

**Fig 9:**
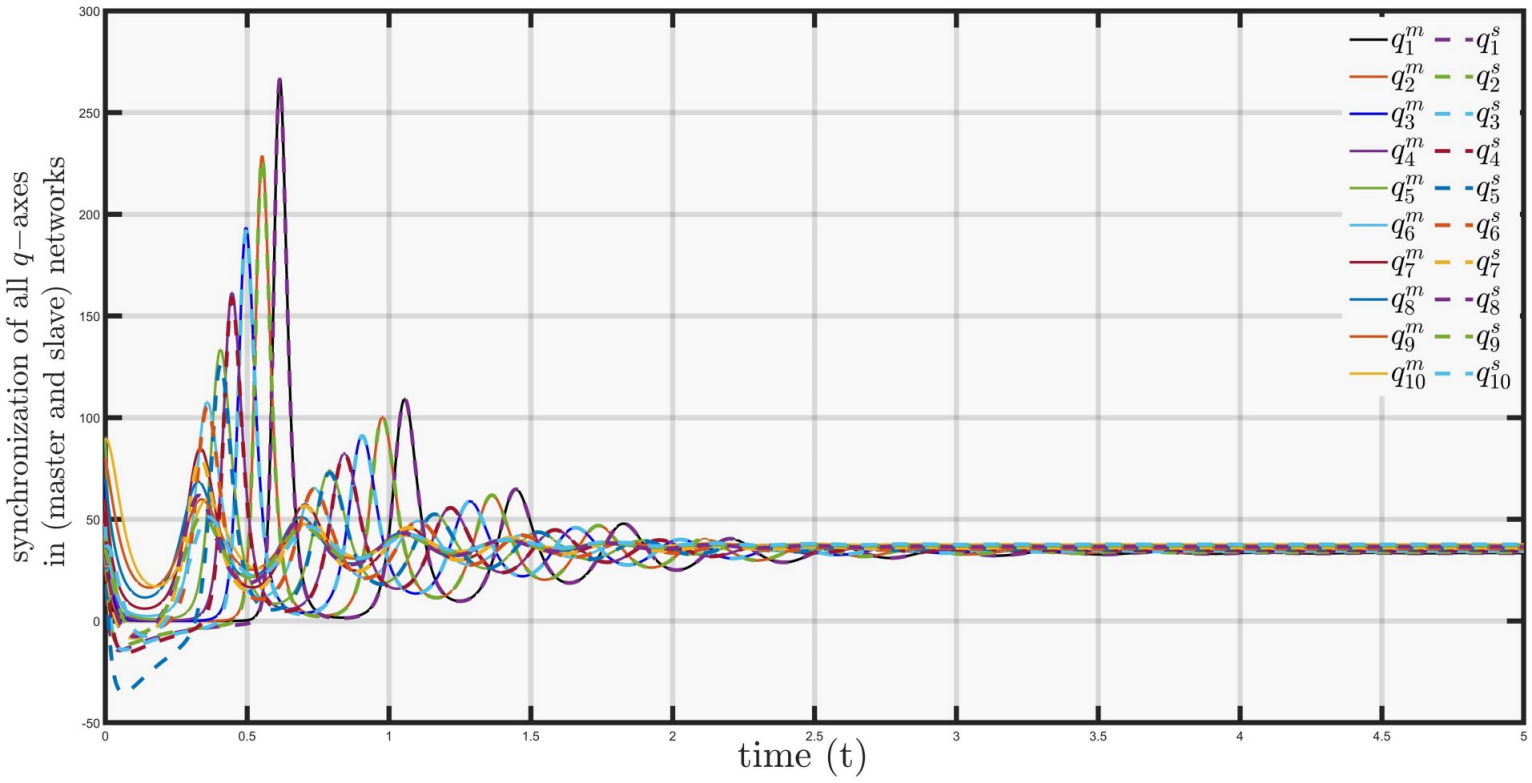
Synchronization of *q*−*axis* in master and slave networks.

In Fig 9 the time history of synchronization of networks based on influenza and covid clusters in *q*−axis can be observed, where the trajectories covid-19 network started following path and adopted the properties of influenza network for *t* = 0.5.

**Fig 10:**
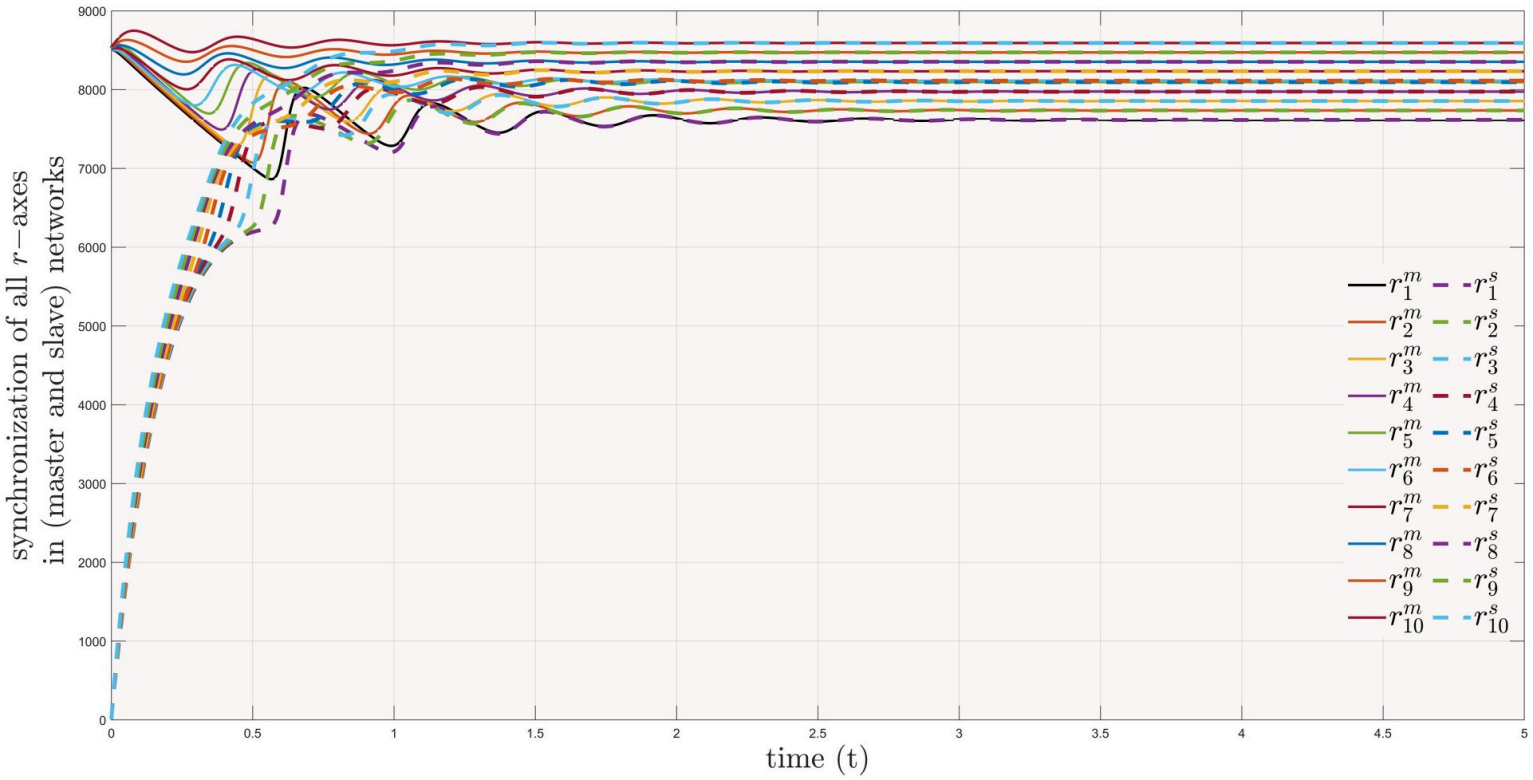
Synchronization of *r*−*axis* in master and slave networks.

**Fig 11:**
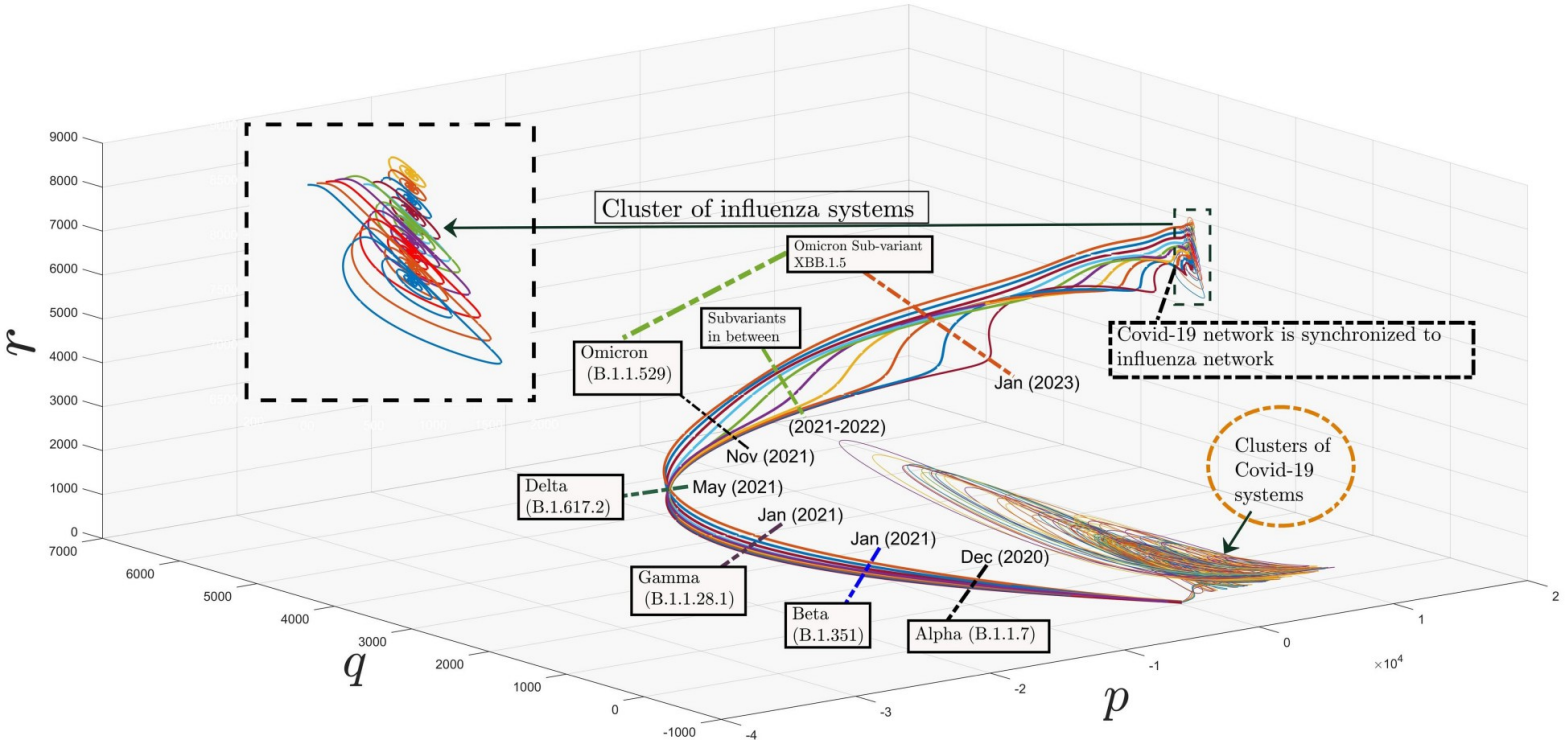
Synchronization of networks based on covid-19 and influenza systems.

Similarly, Fig 10 presents the synchronization of *r*−axis between considered networks. In Fig 11, one can see network of influenza inside a black rectangular shape, which is also zoomed in for more clarification. Similarly, the cluster including covid-19 systems are also plotted using parameter values taken from Table (2). Moreover, one can observe the connection between them, which is the error dynamical system between the trajectories of master and slave networks. Interestingly, the trajectories of covid-19 network are synchronizing towards the network of influenza by passing through several variants. Their WHO, Pango lineage labels, years and variants of concerns (VOC) are written with each variant. It can also be seen in Fig 11 that as the covid-19 passes each variant it is becoming weaker and moving nearer to influenza so that at last there will be a variant, which will become weak, that covid and influenza will be treated equal. From medical point of view, our results verify that, it is also possible to add extra chemical formulae in the pharmacy and medicines of influenza for the treatment of covid-19.

## 4. Conclusion

In this paper, our main aim was to achieve the synchronization between two networks, based on clusters of chaotic systems. We have proved Theorem (2.2) to ensure that as error terms approaches to zero, the trajectories of our considered networks overlaps with each other. We have also included a generalized parameter; *ρ* in error term, which work as a bridge between synchronization and anti-synchronization. But, apart from its applications in physical sciences, we have also showed through Fig 11 that the trajectories of covid will approach to influenza with the passage of time. This idea has been elaborated in view of the emergence of various variants of covid-19. It was observed in our work that as the covid encounters with its variant, the virus become weaker. We have observed from the history of covid-19 that, during its rise, this virus was very deadly and dangerous throughout the world. Then, in 2020 its first variant; Alpha (B.1.17) was discovered in South Africa which was less dangerous in comparison. After that a variety of variants including Beta, Gamma, Delta and Omicron arises in various portions of the world and each time, the upcoming variant was less dangerous. Now-a-days, the most latest sub-variant of Omicron is discovered in January, 2023. This mutation of covid shows resemblance with flu and is not that much dangerous as in the past. That is why, our findings reveals that in the near future its upcoming variants will enter into the region of influenza. This task was achieved with the aid of synchronization of clustered synchronized networks.

## Supporting information

S1 File The folder attached with the current paper for the synchronization of two discussed chaotic clusters. For the convenience of readers, a readme file is attached within the folder for the usage of codes.
(ZIP)
